# Gender Differences in Medical Students’ Self-Assessment: Longitudinal Multi-Cohort Study

**DOI:** 10.2196/91071

**Published:** 2026-07-17

**Authors:** Lennart Handke, Hendrik Friederichs

**Affiliations:** 1 Medical Education Research Group, Medical School OWL, Bielefeld University, Universitätsstraße 25, Bielefeld, 33615, Germany, 49 52110686623

**Keywords:** self-assessment, gender differences, lifelong learning, progress testing, medical education

## Abstract

**Background:**

Self-assessment is a key requirement for lifelong learning in medicine. Evidence from gender-related research indicates that important moderators affecting self-assessment are influenced by gender. Therefore, systematic gender differences in the accuracy of self-assessment may be assumed.

**Objective:**

The present study aimed to examine gender differences in medical students’ self-assessment. Specifically, this study addressed two research questions: (1) Are there systematic gender differences in medical students’ self-assessment accuracy? (2) What is the magnitude of these gender differences when accounting for academic progress and knowledge?

**Methods:**

Medical students from 3 cohorts at Medical School OWL were surveyed in 3 waves between April 2023 and April 2024 during the Progress Test Medicine. Prior to taking the test, students were asked to indicate the percentage of Progress Test Medicine questions that they expected to answer correctly in 5 knowledge areas. Self-assessment accuracy was calculated as the difference between the subjective self-assessment and the objective test score. Linear mixed models were used to analyze the influence of gender on students’ self-assessment accuracy while accounting for academic progress and knowledge.

**Results:**

A total of 165 students with 404 data points participated in this study (269/404, 66.6% women; 135/404, 33.4% men; mean age 21.96, SD 3.61 years). Across all models, women rated themselves significantly less accurately than men (adjusted *P* values ranged from <.001 to .01). The observed gender effect ranged from –6.08 to –3.74 percentage points.

**Conclusions:**

The results indicated systematic gender differences in medical students’ self-assessment in favor of men, with a magnitude comparable to the average knowledge acquired in an entire semester of study. In view of the potentially negative consequences of inaccurate self-assessment, targeted support for realistic self-assessment during medical studies may be particularly beneficial for women.

## Introduction

Continuing professional development requires physicians to regularly update their knowledge and acquire new clinical skills. However, this challenge begins already during undergraduate medical education, where students must also reflect on their knowledge and skills and regulate their learning accordingly (self-regulated learning) [1,2]. Self-regulated learning is defined as a “cyclical process” in which learners analyze their tasks, define goals, plan learning strategies, monitor their performance, and critically reflect on their results [3]. To meet this demand, medical students must be able to assess their knowledge and skills accurately, which is known as self-assessment [4,5]. Good self-assessment is not only essential for self-regulated learning but is also associated with higher academic performance, student motivation, engagement, and self-efficacy [6-8].

Self-assessment is a complex process influenced not only by the observation of one’s own performance, knowledge, and skills but also by self-image and attitudes. Important factors influencing self-assessment include self-confidence and self-esteem, which in turn are influenced by a person’s socialization and demographic variables such as gender [7,9-11]. Evidence from gender-related research indicates that women often have lower self-esteem and less confidence in their own abilities than men. In addition, women tend to attribute their successes to external factors such as favorable circumstances or luck, whereas they attribute failures to personal shortcomings [11]. Considering these gender-specific differences in central moderators of self-assessment, systematic gender differences in the accuracy of self-assessment may be assumed.

A review of the literature on gender differences in medical students’ self-assessment reveals that the topic remains underexplored. For more than two decades, meta-analyses have concluded that gender differences have not been sufficiently investigated to allow for reliable conclusions [8,12,13]. The current evidence on gender differences in self-assessment can be broadly categorized into two central questions: first, whether women and men systematically under- or overestimate themselves and, second, to what extent their self-assessments deviate from external assessments. Regarding the first question, the literature shows that women systematically underestimate their abilities. For men, in contrast, no consistent pattern emerges. The only consistent finding is that men underestimate themselves less often and overestimate themselves more frequently than women [10,11,13-15]. Regarding the second question, it is difficult to draw clear conclusions from the existing literature. Results vary considerably for both women and men, making it impossible to identify a “typical” deviation. Nevertheless, most studies indicate that the differences are overall lower for men, indicating a higher accuracy in their self-assessments [15-17]. Despite the indications of gender-specific differences in self-assessment, the overall evidence remains limited and is characterized by methodological weaknesses.

The present study aimed to examine gender differences in medical students’ self-assessment and, thereby, contributes to closing the research gap discussed above. Specifically, this study addresses the following research questions:

- Are there systematic gender differences in medical students’ self-assessment accuracy?
- What is the magnitude of these gender differences when accounting for academic progress and knowledge?

Using an objective standard of comparison and calculating standardized effect sizes, this study addresses central methodological limitations of previous research.

## Methods

### Study Design

**Figure 1.**
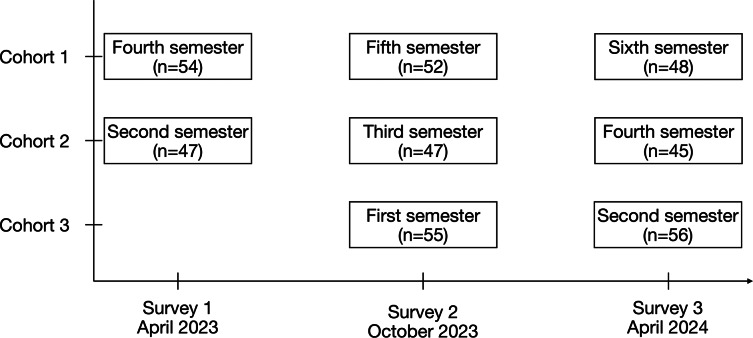
Accelerated longitudinal multi-cohort study design.

To investigate a possible gender bias in medical students’ self-assessment, students from 3 cohorts at Medical School OWL of Bielefeld University were surveyed between April 2023 and April 2024. The duration of undergraduate medical studies in Germany is 6 years. The last year is a practical year in clinical rotations. A total of 3 surveys were conducted during this period. Only students who were enrolled and participated in the required courses were included in this study. To obtain assessments across the first 6 semesters within a single year, an accelerated longitudinal multi-cohort design was implemented. The first cohort took part 3 times (fourth to sixth semester), the second cohort also took part 3 times (second to fourth semester), and the third cohort took part 2 times (first and second semester). The accelerated longitudinal multi-cohort design is illustrated in Figure 1. A priori power analysis was conducted using PowerLMM.js to determine the required sample size for the planned 3-wave measurement structure. We used an α level of .05, a power level of 0.80, an expected effect size of Cohen *d*=0.40, and an anticipated intraclass correlation coefficient of 0.15. On the basis of these assumptions, the required sample size was 336.

### Data Collection and Outcome Measures

The surveys were conducted as part of the Progress Test Medicine (PTM)—developed by Charité in Berlin—a formative assessment consisting of 200 multiple-choice questions in single-best-answer format. The PTM covers the entire medical curriculum at the level of the medical state exam and is administered every 6 months to give the students a regular overview of their progress. The questions are classified by Charité according to subjects, organ systems, domains, and skills, among other aspects.

Prior to taking the test, students were asked to indicate the percentage of the PTM questions that they expected to answer correctly in 5 knowledge areas. The original wording of the self-assessment items (in German) is provided in Multimedia Appendix 1. For this study, a division into subjects (surgical and nonsurgical) and domains (diagnosis, pathogenesis, and therapy) was used. For the differentiation between surgical and nonsurgical subjects, the medical subjects used by Charité to classify the PTM questions were grouped into these 2 superordinate categories (eg, orthopedics, surgery, and urology were classified as surgical and anatomy, internal medicine, and neurology were classified as nonsurgical). The domains were selected from the Charité classification. This division was made to reflect an established differentiation of knowledge in medicine and to have enough PTM items per area to enable statistically valid conclusions. As these are different classification criteria, there are overlaps between the knowledge areas. However, the classification reflects established distinctions in medical education and clinical practice and is therefore familiar to students. An overview of the knowledge areas and the number of associated PTM questions is provided in Multimedia Appendix 1. For this study, the students’ test results were also summarized according to the 5 knowledge areas. As a result, 10 percentage values were calculated for each student: 5 for their self-assessment and 5 reflecting their actual performance in the knowledge areas. Missing data were left as “NA” and handled via listwise exclusion.

To analyze a possible gender bias, the self-assessment accuracy was calculated as the difference between the subjective self-assessment and the objective test score in the various knowledge areas. Positive values indicated an overestimation, and negative values indicated an underestimation. In addition, the absolute difference was used to map the extent of the misjudgments regardless of their direction. To avoid loss of information, self-assessment accuracy was calculated at the individual level, following the methodological recommendations of Ward et al [18].

### Statistical Analysis

A linear mixed model (LMM) was used for the analysis. Gender, academic progress (operationalized as the students’ semester of study), and students’ medical knowledge (total test score) were used as fixed effects (independent variables), and the absolute value of self-assessment accuracy served as the dependent variable. Following the self-assessment literature, academic progress (semester of study) was used as a moderator as it is considered a more appropriate indicator of student experience than age [7,12,19]. Total score was included in the model as previous research has identified student performance as a strong predictor of self-assessment [13,20]. Gender was recorded as “woman,” “man,” and “nonbinary/other.” To account for dependencies within repeated measures, a random intercept for each participant was included in the model. In this way, interindividual differences in the average baseline level of the dependent variable were modeled. Model diagnostics indicated no notable assumption violations. Graphical analysis of residuals and random effects suggested only minor deviations from normality, against which LMMs are considered robust [21]. No indications of heteroscedasticity were observed. The intraclass correlation coefficient exceeded the common threshold of 0.15 and, therefore, supported the use of LMMs over simple regression models [22]. Tests for multicollinearity based on variance inflation factors yielded values of 2.18 or lower, which is clearly below the common cutoff of 5 [23]. The chosen model structure allowed for the examination of both the effects of time-invariant predictors (gender) and time-variant predictors (semester and test score). In addition, LMMs are robust to unbalanced panel structures, such as those present in this dataset [24]. A separate LMM was calculated for each of the 5 knowledge areas. To control for multiple testing, Holm-Bonferroni corrections [25] were applied separately for each independent variable (sex, academic progress, and knowledge level) across the 5 models. This approach reflected the analytical structure of the analysis in which each independent factor represented a statistical family and resulted in 3 statistical families with 5 tests each [26]. All analyses were conducted in R [27] (version 4.5.2; R Foundation for Statistical Computing). The LMMs were created using *lmerTest* [28] and supplemented with the *parameters* [29] and *performance* [30] packages to calculate standardized regression coefficients and model fit indexes.

### Ethical Considerations

Students were informed about the study before completing the test. Participation was voluntary, and students could opt out at any time by skipping individual questions or the self-assessment entirely. Before analysis, all data were anonymized by an independent data trustee at Bielefeld University. For this purpose, personal identifiers in the data were removed and replaced with a randomly generated 10-digit code. The research team only received the anonymized data and had no access to identifying information. This study was approved by the Medical Ethics Committee Westphalia-Lippe (2024-156-f-S) and conducted in accordance with the Declaration of Helsinki.

## Results

Table 1 presents an overview of the sample composition. A total of 90.2% (165/183) of the eligible students volunteered to take part in the study: 33.9% (56/165) from the first cohort, 29.7% (49/165) from the second cohort, and 36.4% (60/165) from the third cohort. This resulted in a total of 404 data points with complete information. The average age of the students at the time of taking the test was 21.96 (SD 3.61) years. In total, 66.6% (269/404) of the participants were women; no one identified as “nonbinary/other.” The gender distribution was typical for medical studies in Germany. According to information from the Federal Statistical Office, 65.5% of medical students across Germany in 2024 were women [31].

**Figure 2.**
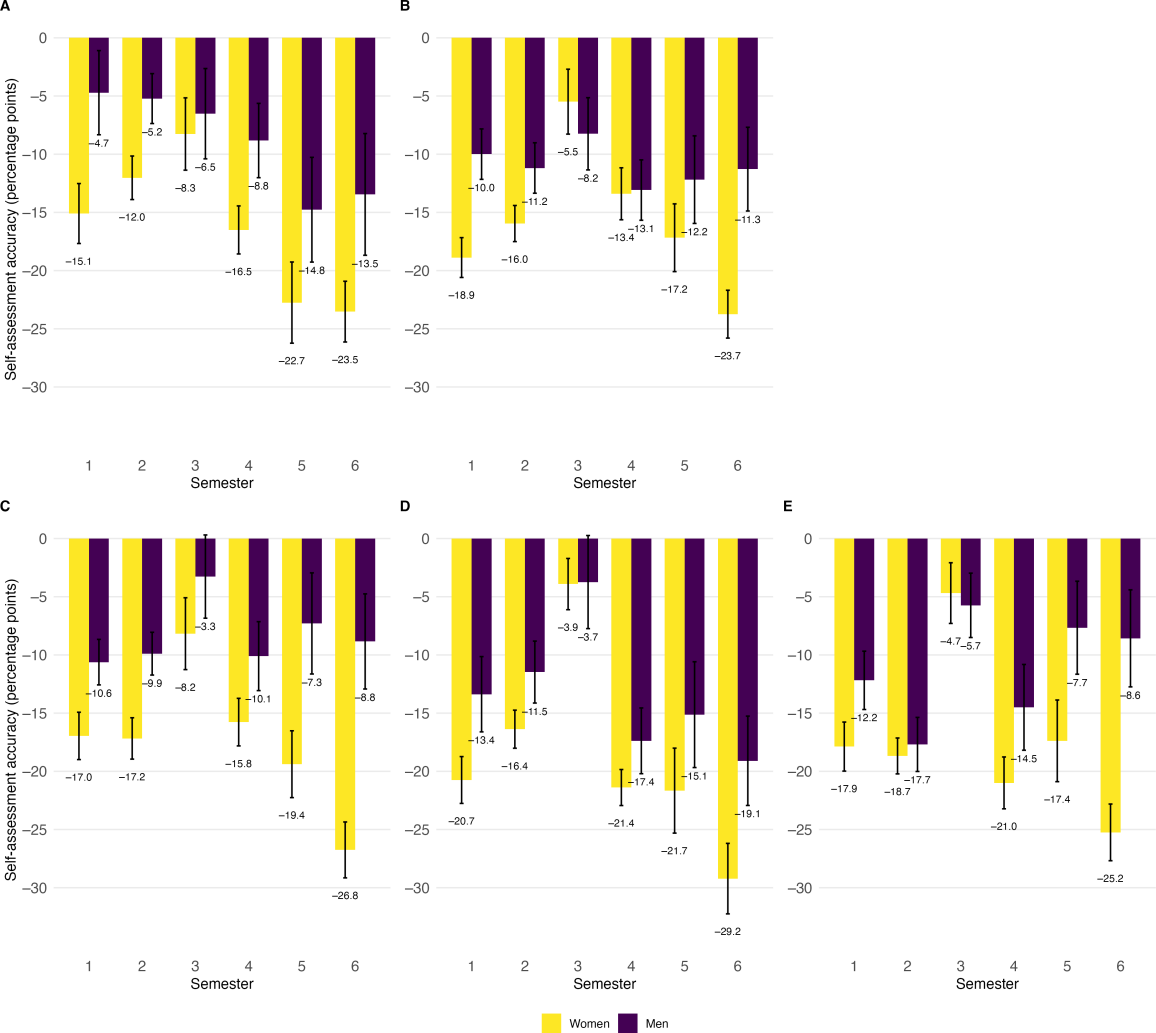
Mean self-assessment accuracy by semester, gender, and knowledge area. Panels show surgical subjects (A), nonsurgical subjects (B), diagnosis (C), therapy (D), and pathogenesis (E). Negative values indicate underestimation; error bars represent SEs.

For an initial examination of whether there were gender-specific differences in students’ self-assessment, the mean values for self-assessment accuracy are presented separately for women and men in Table 2. Figure 2 provides a visual representation of these results to facilitate comparisons across semesters, knowledge areas, and gender groups.

**Table 1.** Age and gender distribution of the study participants.

	All (n=404), n (%)	Men (n=135), n (%)	Women (n=269), n (%)	Age (y), mean (SD)
Semester 1	55 (13.6)	19 (14.1)	36 (13.4)	21.44 (5.12)
Semester 2	103 (25.5)	33 (24.4)	70 (26)	21.15 (3.77)
Semester 3	47 (11.6)	16 (11.9)	31 (11.5)	21.36 (2.25)
Semester 4	99 (24.5)	35 (25.9)	64 (23.8)	22.24 (2.95)
Semester 5	52 (12.9)	17 (12.6)	35 (13)	22.92 (3.3)
Semester 6	48 (11.9)	15 (11.1)	33 (12.3)	23.29 (3.29)
Total	404 (100)	135 (100)	269 (100)	21.96 (3.61)

**Table 2.** Mean value of self-assessment accuracy in the 5 knowledge areas by semester. Negative values indicate underestimation.

Semester	Subjects, percentage points		Domains, percentage points		
	Surgical	Nonsurgical	Diagnosis	Pathogenesis	Therapy
Women					
1	–15.1	–18.88	–16.96	–17.88	–20.75
2	–12.03	–15.96	–17.17	–18.68	–16.38
3	–8.27	–5.49	–8.17	–4.69	–3.91
4	–16.51	–13.4	–15.77	–20.99	–21.39
5	–22.75	–17.18	–19.39	–17.38	–21.66
6	–23.53	–23.74	–26.75	–25.25	–29.22
Men					
1	–4.71	–9.99	–10.62	–12.18	–13.38
2	–5.22	–11.19	–9.89	–17.69	–11.47
3	–6.52	–8.25	–3.27	–5.74	–3.74
4	–8.82	–13.08	–10.1	–14.5	–17.38
5	–14.77	–12.19	–7.29	–7.66	–15.13
6	–13.45	–11.28	–8.84	–8.58	–19.1

Both women and men underestimated themselves in all knowledge areas, but in all except 2 of the 30 subject-by-semester comparisons, women underestimated themselves more than men. The difference between the subjective self-assessment and the objective test score ranged from –29.22 to –3.91 percentage points for women and from –19.1 to –3.27 percentage points for men. Underestimation among women was greatest in the sixth semester (from –29.22 to –23.53 percentage points). The largest difference between men and women was observed in the sixth semester in the area of diagnosis, where women underestimated themselves by 17.91 percentage points more than men. The largest difference in the other direction was in the nonsurgical area in the third semester, where men underestimated themselves by 2.76 percentage points more than women. Among women, underestimation increased over the course of their studies, whereas among men, this pattern was observed only in surgical subjects.

To analyze the extent of self-assessment accuracy in more detail, Table 3 reports absolute differences. In this way, a distortion caused by the offsetting of positive and negative differences is prevented.

**Table 3.** Mean of the absolute self-assessment accuracy in the 5 knowledge areas.

Semester	Subjects, percentage points		Domains, percentage points		
	Surgical	Nonsurgical	Diagnosis	Pathogenesis	Therapy
Women					
1	17.72	19.01	17.11	18.74	20.97
2	16.08	17.53	18.56	19.39	17.6
3	14.65	13.26	15.21	11.24	10.5
4	20.24	19.16	19.56	23.83	22.21
5	27.47	20.1	21.39	22.28	26.26
6	25.55	23.93	27.03	25.8	30.26
Men					
1	13.34	11.75	10.91	14.11	15.69
2	10.17	13.8	12.46	18.1	15.28
3	14.51	12.16	10.89	10.68	12.04
4	16.2	16.22	15.79	20.7	20.49
5	17.33	14.35	13.29	13.95	16.63
6	17.64	12.35	14.65	14.45	19.1

Compared with Table 2, the differences between women and men are smaller in Table 3, mainly because the absolute differences increased more strongly among men. The absolute differences ranged from 10.5 to 30.26 percentage points for women and from 10.17 to 20.7 percentage points for men. Among women, absolute differences also increased over the course of their studies. For men, the absolute differences remained more stable.

The observations from the descriptive analyses were checked using LMMs and examined with regard to significance and effect sizes. The results of the 5 LMMs are shown in Table 4. For the sake of clarity, only the standardized and unstandardized coefficients and *R*^2^ values are reported. The complete model outputs are provided in Multimedia Appendix 1.

**Table 4.** Summary of the fixed effects of the 5 linear mixed models. The reference category for gender is “woman.”

Knowledge area	Gender			Gender, β^a^	Semester			Semester, β	Test score			Test score, β	Marginal *R*^2^	Conditional *R*^2^
	*B* ^b^	Holm-Bonferroni–adjusted *P* value	*P* value		*B*	Holm-Bonferroni–adjusted *P* value	*P* value		*B*	Holm-Bonferroni–adjusted *P* value	*P* value			
Surgical	–4.88	.002	<.001	–0.18	–1.64	.005	.003	–0.21	45.66	<.001	<.001	0.60	0.26	0.452
Nonsurgical	–4.72	.002	<.001	–0.21	–2.52	<.001	<.001	–0.35	41.44	<.001	<.001	0.61	0.216	0.490
Diagnosis	–6.08	<.001	<.001	–0.23	–1.24	.03	.03	–0.16	39.63	<.001	<.001	0.53	0.225	0.493
Pathogenesis	–4.04	.01	.006	–0.15	–2.90	<.001	<.001	–0.35	43.54	<.001	<.001	0.56	0.168	0.351
Therapy	–3.74	.01	.01	–0.13	–2.84	<.001	<.001	–0.34	61.02	<.001	<.001	0.76	0.322	0.527

a Standardized coefficient.

b Unstandardized coefficient.

The marginal *R*^2^, which indicates the variance explained by the fixed effects, ranged from 0.168 (pathogenesis) to 0.322 (therapy). Following Cohen [32], these values are to be regarded as medium (*R*^2^>0.13) to large (*R*^2^>0.26) effect sizes. The conditional *R*^2^, which also takes into account the variance explanation of the random intercept, ranged from 0.351 to 0.527. The difference between the *R*^2^ values shows that a substantial proportion of the variance was due to interindividual differences in the initial level of self-assessment accuracy. Considering standardized coefficients, gender and semester had small (>0.1) to medium (>0.3) effects, and the actual test score had a large effect (>0.5) [32]. However, unstandardized coefficients were more suitable for interpreting the results. Across all models, gender and semester each had negative coefficients, whereas the test score had a positive coefficient. A negative coefficient here indicates a higher accuracy with increasing values of the independent variable. This is consistent with the descriptive analysis, which showed that men assessed themselves more accurately (*B*=–6.08 to –3.74). In the “diagnosis” model, for example, self-assessment and actual test score were 6.08 percentage points further apart for women than for men. In addition, self-assessment became more accurate over the course of medical studies (*B*=–2.90 to –1.24). Students’ actual knowledge showed the largest coefficients across the models (*B*=39.63-61.02), whereby higher test scores were associated with lower self-assessment accuracy. For example, an increase in the test score from 40% to 50% was linked to an average decrease in self-assessment accuracy from 3.96 to 6.1 percentage points. This finding is examined in detail in the Discussion section.

## Discussion

The results of this study show systematic gender differences in medical students’ self-assessment. A consistent pattern emerged: in all LMMs, women rated themselves significantly less accurately than men (adjusted *P* values ranged from <.001 to .01). The difference ranged from –6.08 to –3.74 percentage points (*B*), and the effect sizes ranged from –0.23 to –0.13 (β). Notably, nearly all students underestimated themselves, indicating that these gender differences do not primarily lie in the direction of self-assessment but in the extent of inaccuracy. Thus, the results are generally consistent with the current state of research [11,13,15]. However, it should be emphasized that previous studies have only reported tendencies toward more accurate self-assessment by men. In contrast, the present results showed consistent and significant differences across all models. The magnitude of the gender effects highlights their practical relevance. Considering that medical students typically gain approximately 5% of knowledge in the PTM per semester, the observed difference in self-assessment accuracy between women and men is roughly equivalent to the knowledge acquired in an entire semester [33,34]. Although the effect size of the gender effect is statistically small, it is nevertheless meaningful from an educational perspective.

Another notable finding is the influence of students’ knowledge on their self-assessment accuracy. Contrary to what the current state of research suggests, higher knowledge in this study was associated with lower self-assessment accuracy. This contrasts with previous research, which has generally suggested that self-assessment accuracy improves with increasing knowledge. One possible explanation is that students do not perceive the knowledge they have already acquired as a substantial part of the overall medical curriculum, which may lead to an underestimation of their knowledge level. In addition, as students’ knowledge increases, they may also become more aware of the complexity of the medical curriculum and the limits of their own knowledge. This could lead to a situation in which the standard against which students evaluate their knowledge increases more than their perceived gain in knowledge. This, in turn, may contribute to the observed tendency to underestimate their own performance more strongly despite objectively higher levels of knowledge. In addition to this possible increase in the standard against which students evaluate their knowledge, high-achieving students may hold themselves to an even higher, potentially idealized internal standard. As a result, they may judge their own performance more strictly than lower-achieving students. This could also indicate that students make limited use of available information on their performance (eg, exams and PTM results) in their self-assessment and are therefore unable to adequately assess the extent of their knowledge. According to the model by Yan and Brown [35], independently obtaining and using feedback and information are central elements of self-assessment. Furthermore, ceiling effects may limit further increases in self-assessment for high-achieving students even when their knowledge continues to grow. Moreover, statistical phenomena such as regression toward the mean or other measurement-related artifacts may contribute to the observed counterintuitive influence of students’ knowledge, although the study design was chosen to minimize these risks. However, the present data do not allow for analytical conclusions about the underlying causes, and further research is needed.

Another noteworthy finding of the descriptive analyses is that students’ self-assessment accuracy initially appeared to decrease over the course of their studies (Tables2  3). However, the multivariable results of the LMMs (Table 4) show that academic progress was associated with higher accuracy when knowledge and gender effects were taken into account simultaneously. At the descriptive level, the effect of academic progress was masked by the stronger opposing effect of knowledge. As students’ knowledge increases over the course of their studies and knowledge has a greater effect on self-assessment accuracy than academic progress, the descriptive results suggest that self-assessment becomes less accurate over time. The comparatively small effect of academic progress may also indicate that increasing experience contributes only modestly to improvements in self-assessment and that self-assessment is not systematically developed throughout the curriculum. The observed effect of academic progress is consistent with previous research indicating that self-assessment accuracy tends to improve over time and with increasing student experience.

It is also noteworthy that the medical students assessed their knowledge very imprecisely overall. One possible explanation for this may lie in the design of this study, which uses the entire medical knowledge as a reference standard. Some authors, such as Eva and Regehr [36], argue that people generally have difficulties with realistic self-assessment, especially if the benchmark is abstract and general. The medical curriculum as a whole represents such an abstract benchmark and could therefore partly explain the observed inaccuracy.

Nevertheless, from an analytical standpoint, the use of the PTM is a strength of this study, avoiding some methodological weaknesses common in this field of research. For example, the PTM allows for a longitudinal study as the same benchmark was used for each survey, and the results can therefore be directly compared across time points. As both the self-assessment and the objective comparative measurement were based on the percentage of correctly answered PTM questions, both values could be compared statistically without conversion. As a metric scale, it also allowed for the calculation of effect sizes that can be compared with those of other studies. Another strength of the scale lies in its unambiguous content, which leaves little room for subjective interpretation. Finally, the PTM score is not a subjective evaluation of student performance by teachers but an objective, validated measurement tool. This avoids the difficulties that are often discussed under the term “gold standard” [18,37,38].

A key limitation of this study is that it was a single-center study, so results may differ at other medical faculties (eg, due to differences in curricula or program organization). Generalizations can therefore only be made with caution. However, as progress tests are carried out at many medical faculties, comparable or multicenter studies would be desirable to verify generalizability. In addition, the assignment of PTM items to specific subjects and domains cannot always be considered strictly distinct. Some questions or answer options may address content from multiple disciplines, which may lead students to conceptually associate certain items with a different subject or domain than that specified by the Charité classification. Furthermore, the number of PTM items assigned to each subject or domain varies slightly between test administrations. As a consequence, percentage scores are not equally stable across all subjects because individual items carry greater weight in subjects with fewer questions. To mitigate these limitations, individual subjects were aggregated into superordinate knowledge areas, thereby reducing the influence of single items and buffering minor ambiguities in content classification. Furthermore, this study did not assess additional variables that could help explain the observed gender differences in self-assessment. The underlying mechanisms of these differences could therefore not be examined directly.

In view of the potentially negative consequences of inaccurate self-assessment (as described in the Introduction section), targeted support for realistic self-assessment during medical studies may be particularly beneficial for women. Implications for curriculum design could include, for example, targeted feedback, training to improve self-assessment accuracy, and gender-sensitive teaching interventions. However, as the self-assessment was inaccurate not only among women but across all students, additional measures should be implemented to help students situate their performance within the broader context of their studies. Possible approaches include regular feedback formats, transparent performance evaluations, and guided opportunities for reflection. To sustainably reduce gender disparities in self-assessment, however, it is not sufficient to implement measures exclusively at the student level. Educators, evaluators, and faculty members should also be made aware of potential gender differences and trained in how to address them. For this purpose, it would be beneficial to regularly evaluate existing gender differences at the faculty level and make them transparent to faculty members and relevant committees. Furthermore, potential causes of gender disparities, including university-level factors such as teaching, examination, and feedback formats, as well as specific strategies for reducing them, should be addressed in structured professional development programs. Such measures should be institutionally anchored at the faculty level and integrated into the curriculum to ensure that their implementation does not depend solely on the commitment of individual educators.

For future research, it would be worthwhile to analyze the mechanisms underlying the observed gender differences in more detail. In particular, studies should examine how women and men differ in their interpretation and use of performance-related feedback and how these differences contribute to the observed gender bias in students’ self-assessment. In addition, future research should examine how teaching, examination, and feedback practices may unconsciously contribute to the emergence and maintenance of the observed gender differences. At the institutional level, future research should also investigate whether gender-related stereotypes or expectations are conveyed in medical studies in ways that may contribute to the development of such differences. With regard to the counterintuitive influence of students’ knowledge on self-assessment accuracy, future research should investigate how students perceive the medical curriculum and conceptualize their own knowledge within it. Furthermore, future studies should explicitly model potential sociocultural factors such as achievement motivation and internalized gender roles to examine their contribution to the observed gender bias.

## Supplementary material

10.2196/91071 Multimedia Appendix 1 Supplementary tables detailing the English translation of the self-assessment items, the composition of the knowledge areas based on the subject and domain classification used by Charité, the number of questions per area, and the complete linear mixed model results.

## Abbreviations

LMM: linear mixed model
PTM: Progress Test Medicine
