# Supplementary material for: Gender Differences in Medical Students’ Self-Assessment: Longitudinal Multi-Cohort Study
Source: JMIR Med Educ. 2026 Jul 17;12:e91071. doi: 10.2196/91071 (PMC13378408; doi:10.2196/91071)
Supplement: Multimedia Appendix 1 [file mededu-v12-e91071-s001.docx]

# Multimedia Appendix

| Question | Answer option |
| --- | --- |
| What percentage of the questions from **surgical subjects** (e.g., surgery, gynecology, ophthalmology, …) do you think you will be able to answer correctly? | Free-text response |
| What percentage of the questions from **non-surgical subjects** (e.g., internal medicine, hygiene, pediatrics, …) do you think you will be able to answer correctly? | Free-text response |
| What percentage of the questions from the **diagnosis** domain do you think you will be able to answer correctly? | Free-text response |
| What percentage of the questions from the **therapy** domain do you think you will be able to answer correctly? | Free-text response |
| What percentage of the questions from the **pathogenesis** domain do you think you will be able to answer correctly? | Free-text response |

Table A1: English translation of the wording of the self-assessment questions used in the study. The original items were administered in German.

| Knowledge area | Included subjects / domains of the Charité | |
| --- | --- | --- |
| Surgical subjects | Dermatology, Venereology | Otorhinolaryngology |
|  | Gynecology and Obstetrics | Surgery |
|  | Ophthalmology | Urology |
|  | Orthopaedics |  |
| Non-surgical subjects | Anatomy | Molecular Biology |
|  | Anesthesiology, Emergency and Intensive Care Medicine | Naturopathy, Physical Medicine |
|  | Biochemistry | Neurology |
|  | Biology | Occupational and Social Medicine, Health Care |
|  | Chemistry | Pathology |
|  | Clinical Chemistry, Laboratory Diagnostics | Pediatrics |
|  | Epidemiology, Medical Biometry | Pharmacology, Toxicology |
|  | Forensic Medicine | Physics |
|  | General Medicine | Physiology |
|  | Human Genetics | Psychiatry |
|  | Hygiene, Microbiology | Psychosomatics |
|  | Internal Medicine | Radiology, Nuclear Medicine |
|  | Medical Psychology/Sociology |  |
| Diagnosis | Diagnosis Diagnostics | |
| Pathogenesis | Pathogenesis Pathomechanisms | |
| Therapy | Therapy | |

Table A2: Composition of the knowledge areas.

| Survey | Surgical | Non-surgical | Diagnosis | Pathogenesis | Therapy |
| --- | --- | --- | --- | --- | --- |
| Survey 1 | 42 | 158 | 63 | 41 | 31 |
| Survey 2 | 46 | 154 | 100 | 39 | 29 |
| Survey 3 | 33 | 167 | 84 | 55 | 14 |

Table A3: Number of questions per knowledge area.

| Predictor | *B* | 95% CI (*B*) | β (Std. Coef.) | 95% CI (β) | *t* | df | Pr(>\|*t*\|)  *P*_adj_ |
| --- | --- | --- | --- | --- | --- | --- | --- |
| (Intercept) | 7.47 | [4.36, 10.57] | - | - | 4.701 | 282.03 | < .001  < .001 |
| Gender | -4.88 | [-7.53,  -2.23] | -0.18 | [-0.28,  -0.08] | -3.602 | 160.07 | < .001  .002 |
| Semester | -1.64 | [-2.69,  -0.58] | -0.21 | [-0.34,  -0.07] | -3.045 | 308.86 | .003  .005 |
| Score | 45.66 | [35.54, 55.77] | 0.60 | [0.47, 0.74] | 8.811 | 302.56 | < .001  < .001 |

Table A4: LMM surgical subjects

*P*_adj_ = Holm-Bonferroni adjusted *P*-Value. Conditional *R*² = 0.452; Marginal *R*² = 0.260. Random intercept variance (ID): 30.56; Residual variance: 87.15. 95% CIs for unstandardized coefficients computed via profile likelihood. Standardized coefficients computed via model refitting.

| Predictor | *B* | 95% CI (*B*) | β (Std. Coef.) | 95% CI (β) | *t* | df | Pr(>\|*t*\|)  *P*_adj_ |
| --- | --- | --- | --- | --- | --- | --- | --- |
| (Intercept) | 11.12 | [8.17, 14.06] | - | - | 7.369 | 300.44 | < .001  < .001 |
| Gender | -4.72 | [-7.31,  -2.12] | -0.20 | [-0.31,  -0.09] | -3.550 | 165.66 | < .001  .002 |
| Semester | -2.52 | [-3.52,  -1.52] | -0.35 | [-0.49,  -0.21] | -4.966 | 331.41 | < .001  < .001 |
| Score | 41.44 | [31.81, 51.00] | 0.61 | [0.47, 0.75] | 8.470 | 329.87 | < .001  < .001 |

Table A5: LMM non-surgical

*P*_adj_ = Holm-Bonferroni adjusted *P*-Value. Conditional *R*² = 0.49; Marginal *R*² = 0.216. Random intercept variance (ID): 36.15; Residual variance: 67.29. 95% CIs for unstandardized coefficients computed via profile likelihood. Standardized coefficients computed via model refitting.

| Predictor | *B* | 95% CI (*B*) | β (Std. Coef.) | 95% CI (β) | *t* | df | Pr(>\|*t*\|)  *P*_adj_ |
| --- | --- | --- | --- | --- | --- | --- | --- |
| (Intercept) | 8.62 | [5.26, 12.01] | - | - | 5.131 | 292.30 | < .001  < .001 |
| Gender | -6.08 | [-8.96,  -3.20] | -0.23 | [-0.34,  -0.12] | -4.124 | 156.23 | < .001  < .001 |
| Semester | -1.24 | [-2.35,  -0.13] | -0.16 | [-0.30,  -0.02] | -2.177 | 326.01 | .03  .03 |
| Score | 39.63 | [28.70, 50.43] | 0.53 | [0.39, 0.68] | 7.243 | 323.01 | < .001  < .001 |

Table A6: LMM diagnosis

*P*_adj_ = Holm-Bonferroni adjusted *P*-Value. Conditional *R*² = 0.493; Marginal *R*² = 0.225. Random intercept variance (ID): 44.08; Residual variance: 83.68. 95% CIs for unstandardized coefficients computed via profile likelihood. Standardized coefficients computed via model refitting.

| Predictor | *B* | 95% CI (*B*) | β (Std. Coef.) | 95% CI (β) | *t* | df | Pr(>\|*t*\|)  *P*_adj_ |
| --- | --- | --- | --- | --- | --- | --- | --- |
| (Intercept) | 13.35 | [9.77, 16.89] | - | - | 7.719 | 268.16 | < .001  < .001 |
| Gender | -4.04 | [-6.87,  -1.21] | -0.15 | [-0.25,  -0.04] | -2.781 | 151.00 | .006  .01 |
| Semester | -2.90 | [-4.01,  -1.69] | -0.35 | [-0.49,  -0.21] | -4.896 | 295.97 | < .001  < .001 |
| Score | 43.54 | [32.48, 54.61] | 0.56 | [0.41, 0.70] | 7.675 | 285.53 | < .001  < .001 |

Table A7: LMM pathogenesis

*P*_adj_ = Holm-Bonferroni adjusted *P*-Value. Conditional *R*² = 0.351; Marginal *R*² = 0.168. Random intercept variance (ID): 30.91; Residual variance: 110.15. 95% CIs for unstandardized coefficients computed via profile likelihood. Standardized coefficients computed via model refitting.

| Predictor | *B* | 95% CI (*B*) | β (Std. Coef.) | 95% CI (β) | *t* | df | Pr(>\|*t*\|)  *P*_adj_ |
| --- | --- | --- | --- | --- | --- | --- | --- |
| (Intercept) | 6.90 | [3.64, 10.16] | - | - | 4.144 | 286.12 | < .001  < .001 |
| Gender | -3.74 | [-6.55,  -0.94] | -0.13 | [-0.23,  -0.03] | -2.602 | 156.68 | .01  .01 |
| Semester | -2.84 | [-3.95,  -1.72] | -0.34 | [-0.47,  -0.21] | -5.056 | 315.38 | < .001  < .001 |
| Score | 61.02 | [50.11, 71.79] | 0.76 | [0.63, 0.90] | 11.290 | 310.88 | < .001  < .001 |

Table A8: LMM therapy

*P*_adj_ = Holm-Bonferroni adjusted *P*-Value. Conditional *R*² = 0.527; Marginal *R*² = 0.322. Random intercept variance (ID): 38.41; Residual variance: 88.36. 95% CIs for unstandardized coefficients computed via profile likelihood. Standardized coefficients computed via model refitting.
